# When sexual threat cues shape attitudes toward immigrants: the role of insecurity and benevolent sexism

**DOI:** 10.3389/fpsyg.2015.01033

**Published:** 2015-07-28

**Authors:** Oriane Sarrasin, Nicole Fasel, Eva G. T. Green, Marc Helbling

**Affiliations:** ^1^Migration and Diversity, WZB Berlin Social Science CenterBerlin, Germany; ^2^National Centre of Competence in Research LIVES, Faculty of Social and Political Sciences, University of LausanneLausanne, Switzerland; ^3^Institute of Psychology, Faculty of Social and Political Sciences, University of LausanneLausanne, Switzerland; ^4^Department of Political Science, University of BambergBamberg, Germany

**Keywords:** threat, prejudice, immigration, sexual violence, insecurity, benevolent sexism

## Abstract

Drawing on psychological and political science research on individuals’ sensitivity to threat cues, the present study examines reactions to political posters that depict male immigrants as a sexual danger. We expect anti-immigrant attitudes to be more strongly predicted by feelings of insecurity or representations of men and women as strong and fragile when individuals are exposed to sexual threat cues than when they are not. Results from two online experiments conducted in Switzerland and Germany largely confirmed these assumptions. Comparing two anti-immigrant posters (general and non-sexual threat vs. sexual threat), Experiment 1 (*n* = 142) showed that feelings of insecurity were related to an increased support for expelling immigrants from the host country in both cases. However, only in the sexual threat cues condition and among female participants, were perceptions of women as fragile—as measured with benevolent sexism items—related to support for expelling immigrants. Further distinguishing between different forms of violence threat cues, Experiment 2 (*n* = 181) showed that collective feelings of insecurity were most strongly related to support for expelling immigrants when a male immigrant was presented as a violent criminal. In contrast, benevolent sexist beliefs were related to anti-immigrant stances only when participants were exposed to a depiction of a male immigrant as a rapist. In both cases attitudes were polarized: on the one hand, representations of immigrants as criminals provoked reactance reactions—that is, more positive attitudes—among participants scoring low in insecurity feelings or benevolent sexism. On the other hand, those scoring high in these dimensions expressed slightly more negative attitudes. Overall, by applying social psychological concepts to the study of anti-immigrant political campaigning, the present study demonstrated that individuals are sensitive to specific threat cues in posters.

## Introduction

Discriminatory attitudes and acts against immigrants are rife in many Western countries. A recent report from the OECD (2013), for instance, states that in these countries up to 15% of first-generation immigrants and 25% of second-generation immigrants feel that they belong to a discriminated group. Anti-immigrant attitudes are in great part explained by the fact that immigration is often described and perceived as a threat to the receiving country (Wagner et al., 2010). Perceptions of immigrants threatening the local economy (e.g., by “stealing” jobs or abusing social benefits) and culture (e.g., by having different religious or cultural values and practices) are widespread. Numerous studies have been devoted to investigating the antecedents and consequences of economic and symbolic threats related to immigration (Green et al., 2015). In contrast, the impact of another form of threat, that is perceiving male immigrants as a sexual danger, has attracted far less research attention. This lack of interest is surprising, since male out-group members—in particular men from ethnic and migrant minorities—are generally perceived as more dangerous and violent than their female counterparts (Quillian and Pager, 2001; Plant et al., 2011). In addition, compared to in-group men, out-group men are more often seen as a sexual danger to in-group women (Nagel, 2001; Navarrete et al., 2010). Echoing and maybe galvanizing these fears, information and messages associating male immigrants with sexual violence are frequently found in the media. For example, when analyzing media reports of sexual offenses in the UK, Grover and Soothill (1996) found that offenders’ ethnicity was more often mentioned when they had an ethnic or immigrant background than when they did not. Radical right parties have also suggested that male immigrants can be a danger to women. As an illustration, a few years ago, the streets of Switzerland were plastered with a poster featuring “Ivan,” a fictitious male immigrant character—most likely from Eastern Europe—convicted of rape and on the verge of obtaining Swiss citizenship.

Drawing on psychological and political science research on individuals’ sensitivity to threat cues, the present study examines reactions to political posters that associate male immigrants with sexual violence. We expect that threatening representations of immigrants are most effective when their content resonates with specific antecedents of anti-immigrant attitudes. Based on this reasoning, sexual threat cues should influence to a greater extent the attitudes of those who fear violence or who believe that men’s duty is to protect women. In contrast, those who do not adhere to such beliefs may reject the messages conveyed in posters containing sexual threat cues, and consequently, out of reactance, express more positive views. These assumptions were tested and largely confirmed in two online experiments. The first experiment—conducted in Switzerland (*n* = 142)—compared two real anti-immigrant posters (i.e., previously used in political campaigning) representing immigrants, or a subcategory of them, either as a general threat or as a sexual threat. To confirm Experiment 1’s findings, a second experiment was conducted with fictitious posters in a comparable national setting where this type of poster was unknown (Germany; *n* = 181). Moreover, to disentangle the violence aspects from the gendered aspects contained in sexual threat cues, we extended our investigation by comparing reactions to different representations of criminal immigrants (i.e., as a rapist, a violent criminal, or a drug dealer).

### Threat Cues

Members from the national majority may feel threatened by immigration based on what they observe in their everyday lives. However, their primary source of information on immigration and immigrants is generally indirect, such as through the media (Blumer, 1958; Brader et al., 2008), in which ethnic and migrant minorities tend to be overrepresented and linked to negative events (van Dijk, 1991; Lubbers et al., 1998). Political parties also often mention immigration in their advertisements, which is, in general, an effective way of increasing voters’ knowledge about specific issues (Groenendyk and Valentino, 2002; Holbert et al., 2002). Negative attitudes toward immigrants and immigration are a key antecedent of support for radical right parties (Ivarsflaten, 2008); it is thus unsurprising that these parties in particular often draw on threatening representations of immigrants to mobilize voters (e.g., in Switzerland, Garufo and Maire, 2013). Indeed, when fear is triggered, vigilance tends to rise and individuals are more easily persuaded (Brader, 2005).

Because visual imagery—and especially when conveying threatening representations—is a powerful means to affect political and social attitudes (Huddy and Gunnthorsdottir, 2000; Maire, 2015), political parties’ primary way of communicating—especially in Europe—is through posters, while TV ads are more often used in the US (Holtz-Bacha and Kaid, 2006). When walking down a street or driving, individuals have only a few seconds to catch the meaning of a political poster. Thus, messages need to be strong and easy to catch (Lessinger et al., 2003), such as in the much-debated posters used by the Swiss Peoples’ Party, the main Swiss radical right party. A famous example is a poster used during the campaign preceding a referendum on the expulsion of criminal immigrants, in which white sheep standing on the Swiss flag kick out a black sheep.

It is very likely that radical right parties’ anti-immigrant posters have a greater impact on some individuals than on others. Indeed, social psychological research has shown that representations of out-group members as threatening activate prejudice among those predisposed to it, because of the fears they have, the ideologies they hold or their attachment to their group (e.g., Brewer, 1999; Jackson, 2002). Thus, threatening depictions of immigrants—or even their mere presence—have been found to have a stronger effect on those predisposed to reject immigration. For instance, the highest rejection of immigrants was found in individuals afraid of walking alone at night when they lived in areas with a high immigration rate (Sibley et al., 2013). Similarly, a meta-analysis by Cohrs and Stelzl (2010) revealed that the relationship between believing in norms, traditions and the power of authorities, and negative attitudes toward immigrants is particularly strong in countries where immigrants are perceived to increase the crime rate. Individuals holding these beliefs were also found to react with harsher immigration attitudes when immigrants were presented as adhering to different norms and values (Duckitt and Sibley, 2010) or as rejecting the local norms (and, by doing so, breaching in-group conformity; Thomsen et al., 2008).

While this “activating” effect of threatening representations is currently a topic of interest in social psychology, in other fields only two studies have, to our knowledge, sought to explain individuals’ sensitivity to anti-immigrant political posters. Both focusing on educational attainment, these two studies confirmed that threat cues are more effective when their content resonates with specific antecedents of prejudice. In a study conducted in Switzerland, Matthes and Marquart (2013) assumed that those with low education are not only more likely to feel in competition with immigrants for the same goods (e.g., jobs, housing, and benefits) but, because they are less used to generating counterarguments when faced with political messages, they should also be more easily persuaded by these messages. Confirming these assumptions, these authors found that when seeing an anti-immigrant poster with a strong emotional message, individuals with low education reported fearing immigrants to a greater extent than those with high education. Highly educated people, in contrast, had an aversive (or “reactance”) reaction, by expressing the most negative stances toward radical right campaigning when exposed to the same poster. However, because they did not distinguish between different types of threat, it was not possible to determine whether perceived economic threat or persuasion explained the sensitivity of those with low education. Comparing different immigration-related threats in a study conducted in Austria, Schmuck and Matthes (2014) confirmed the role played by perceived economic competition. After exposure to a poster that pictured immigrants as an economic threat, only adolescents with low educational attainment became more negative toward immigration. In contrast, the impact of education on anti-immigrant attitudes was not moderated by a depiction of immigrants as threatening the host nation’s culture (compared to adolescents with similar educational attainment in a “no poster” condition). In the present study we apply this “sensitivity to threat cues” reasoning to the case of representations of male immigrants as a sexual threat. Because physical violence and gender relations are two core aspects of sexual violence, we assume that threat depictions inducing these aspects will affect most those who fear violence (i.e., who report feelings of insecurity) or who believe in the protective role of men (i.e., who express benevolent sexist beliefs). Conversely, it may be that individuals who score low on these two dimensions reject the messages conveyed by the political posters, and, as a consequence, express more positive attitudes toward immigrants.

### Feelings of Insecurity

Because presenting male immigrants as a sexual danger relies on the stereotypical view that immigrants are dangerous and violent, sexual violence cues are assumed to most influence those who feel concerned about security. While a clear association between immigration and crime is far from established (Hiatt, 2007), immigrants are indeed often associated with crime and violence, in radical right parties’ messages (e.g., Maire, 2015) as well as in individuals’ representations (e.g., Quillian and Pager, 2001; Dinas and van Spanje, 2011). Individuals can fear for their own security, or for security in their community (Schwartz and Boehnke, 2004). Feelings of personal insecurity relate to fears and worries for one’s own well-being and safety, while feelings of collective insecurity imply fearing that the stability and harmony of larger entities (e.g., cities, nations) is at stake. Both are known to influence individuals’ attitudes toward immigrants: fearing for one’s own security, such as when walking alone in one’s neighborhood after dark, has been related to negative attitudes toward immigrants (e.g., Chandler and Tsai, 2001; Rustenbach, 2010), whereas feelings of collective insecurity, such as worrying about crime, have been found to predict concerns about immigration more than the actual crime rate (Fitzgerald et al., 2012). Based on these studies, we expect that feelings of both personal and collective insecurity underlie negative attitudes toward immigrants. When encountering a poster associating male immigrants to sexual violence, compared to an anti-immigrant poster where sexual threat cues are absent, it is particularly those who feel insecure who should react with harsher anti-immigrant attitudes. Because collective insecurity pertains to society as a whole, we expect men and women with strong feelings of collective insecurity to react in a similar way when facing sexual threat cues. In contrast, when exposed to depictions of a male immigrant as a rapist, the impact of personal security should be stronger for women, the primary targets of sexual violence, than for men.

### Benevolent Sexist Beliefs

Sexual violence cues are also assumed to have the strongest influence on those who believe that—weaker but complementary to men—women need to be protected. Based on representations of men and women as, respectively, strong and fragile, these beliefs are part of what is called benevolent sexism, one of the two aspects of ambivalent sexism. Indeed, attitudes toward women are known to be quite complex: Across cultures, hostile sexism—a unidimensional construct—has been distinguished from the more complex benevolent sexism (Glick and Fiske, 1996; Glick et al., 2000). On the one hand, women violating traditional gender roles, such as feminists or career women, tend to face blatant intolerant attitudes. These openly negative attitudes define hostile sexism. On the other hand, attitudes toward women generally are often more positive than those toward men. This can be explained by the prevalence of the beliefs that women are “better” (e.g., purer, more sensitive) human beings than men. These seemingly positive attitudes nevertheless rely on negative stereotypical views of women: they are seen as fragile and requiring protection from men, who, by way of contrast, are perceived as much stronger. These beliefs—complementary gender differentiation (CGD) and protective paternalism (PP)—are two components of benevolent sexism that pertain to the strength vs. fragility perceived opposition between men and women. The third component of benevolent sexism—heterosexual intimacy—is defined as “a genuine desire for psychological closeness” (Glick and Fiske, 1996, p. 493).

Despite their differences, hostile and benevolent sexism are generally positively correlated, because they both rely on stereotypical views of women (Glick and Fiske, 1996; Sibley and Perry, 2010). We thus expect this to be the case here. In turn, since they both entail blatant negative attitudes expressed toward disadvantaged groups, hostile sexism and attitudes toward immigrants should covary (such as “traditional” sexism and racism are generally found to do; e.g., Aosved and Long, 2006; Zick et al., 2008). In contrast, because of its insidious but subtle nature, benevolent sexism should be only weakly related to overt anti-immigrant stances, and this especially when hostile sexism is also considered. In line with this assumption, benevolent sexism, when controlling for hostile sexism, has been found to be unrelated to the endorsement of blatant hierarchies between social groups, a general ideological tendency known to predict different forms of prejudice, such as anti-immigrant stances (Christopher and Mull, 2006). However, when women’s alleged weakness and need for protection are made salient by depictions of male immigrants as rapists, individuals associating strength to men and fragility to women (i.e., scoring high in CGD and PP) are likely to react with negative attitudes toward immigrants. We expect to find this effect in both men and women. On the one hand, for men scoring high on these two dimensions the willingness to protect women from the alleged threat (i.e., the male immigrant aggressor) is increased. For this reason, they are likely to express heightened negative attitudes toward immigrants. On the other hand, presenting immigrants as a sexual threat is likely to activate the need to be protected among women with strong benevolent sexist beliefs. Women with such beliefs may thus express negative immigration attitudes after exposure to cues that render salient the aggressive potential of some men—here immigrants. Finally, we do not expect the impact of heterosexual intimacy to be activated by sexual threat cues because this dimension of benevolent sexism pertains above all to personal relationships.

## Experiment 1

### Method

#### Participants

Our online questionnaire, conducted with Psychsurveys.org, targeted the French-speaking region in Switzerland. Generally, there is evidence that findings drawn from online samples are consistent with those obtained with traditional methods (Gosling et al., 2004). Moreover, we relied on data collected online because they allow more extrapolation to the general population than, for instance, student samples. Recruiting participants from all social backgrounds is all the more important in the present case because individuals with low and high educational attainment are known to react differently to anti-immigrant posters (Matthes and Marquart, 2013; Schmuck and Matthes, 2014). Snowball sampling was used to collect the data: the first author contacted a large number of acquaintances by email, to invite them to both take part in the study and forward the invitation email. Four hundred and sixty-two individuals clicked on the link, and 181 completed the questionnaire. From the final sample, only 27.54% indicated having received the invitation directly from the first author (note that participants who were contacted directly by the first author did not differ significantly in any of the measures of interest from those who were not).

We selected data of Swiss citizens (*n* = 142; 83 women and 59 men). Participants ranged in age from 19 to 100 (Median = 30, Mean = 34.31, SD = 13.62). Forty-five respondents reported having another citizenship in addition to Swiss, and slightly more than two thirds of the sample (*n* = 102) had a high school diploma.

#### Procedure

Participants first completed a series of scales on various “societal topics” (as described in the questionnaire), including items measuring feelings of insecurity and sexist beliefs. Then, they read that the second part of the questionnaire pertained to immigration specifically, as a central topic to many recent political campaigns in Switzerland. To capture the participants’ attention, we first presented to all of them a poster that was seen in the streets of Switzerland at the time we launched our survey. Used in a campaign to reduce migration to Switzerland, the poster showed feet walking on the Swiss flag, accompanied with the “Stop mass immigration” slogan. On the same page, participants also saw one of two posters used during a slightly older campaign that preceded a referendum on the expulsion of criminal immigrants (in November 2009). They were randomly assigned to one of the two conditions by Psychsurveys. In the sexual threat condition (*n* = 73) participants were presented the Ivan poster. This poster showed a fictitious immigrant character—most likely of Balkan or Eastern European origin—convicted of rape and on the verge of obtaining Swiss citizenship. “Ivan, rapist, and soon to be Swiss?” was written in a large black bar hiding part of his face. In the non-sexual threat condition (*n* = 69), they were presented the Sheep poster, in which three white sheep are standing on the Swiss flag as one of them kicks a black sheep away. These posters are very well-known in Switzerland: Only six participants in the sexual threat (Ivan) condition, and no one in the non-sexual threat (Sheep) condition reported having never seen the poster. Finally, participants were invited to respond to various measures related to immigration, including the dependent variable of the present study.

This study was carried out in accordance with the recommendations of both the Code of Deontology of the Swiss Psychological Society and the American Psychological Association. Before completing the questionnaire, participants were informed that they were about to take part in a scientific study and that their responses would remain anonymous. They were also told that they could pass a question or quit the survey at any time. In the months following their participation in the study, participants received an email describing the goals and results of the study.

#### Measures

The whole questionnaire was in French. Means, SD, and score intercorrelations are displayed in **Table 1**.

**Table 1:** Means, SD, and intercorrelations of attitudinal variables in Experiments 1 and 2.

Experiment 1	M	SD		BS: PP–CGD	BS: HI	HS	EXP
Insecurity	2.51	(2.49)		0.22^∗∗^	0.19^∗∗^	0.26^∗∗^	0.53^∗∗∗^
Benevolent sexism: PP–CGD	2.54	(1.07)			0.52^∗∗∗^	0.57^∗∗∗^	0.45^∗∗∗^
Benevolent sexism: HI	2.69	(1.38)				0.46^∗∗∗^	0.43^∗∗∗^
Hostile Sexism	2.77	(1.27)					0.63^∗∗∗^
Expelling immigrants (EXP)	2.93	(1.66)					

**Experiment 2**	***M***	**SD**	**I-C**	**BS: PP–CGD**	**BS: HI**	**HS**	**EXP**

Insecurity: personal (I-P)	2.44	(0.75)	0.41^∗∗∗^	0.23^∗∗^	0.07	0.21^∗∗^	0.23^∗∗^
Insecurity: collective (I-C)	3.16	(1.62)		0.24^∗∗∗^	0.14^∗^	0.40^∗∗∗^	0.56^∗∗∗^
Benevolent sexism: PP–CGD	3.06	(1.25)			0.63^∗∗∗^	0.50^∗∗∗^	0.38^∗∗∗^
Benevolent sexism: HI	3.75	(1.76)				0.44^∗∗∗^	0.35^∗∗∗^
Hostile Sexism	2.89	(1.17)					0.53^∗∗∗^
Expelling immigrants (EXP)	2.95	(1.86)					

*^∗^p < 0.05, ^∗∗^p < 0.10, ^∗∗∗^p < 0.001.*

Our dependent variable—negative attitudes toward immigrants—was measured by participants’ support for expelling law- and norm-breaking immigrants, since this was the object of both the Ivan and the Sheep posters. To this end, participants were invited to state to what extent—from 1 = *strongly disagree* to 7 = *strongly agree*—they thought that immigrants who were long-term unemployed, were sentenced to prison or were illegally in Switzerland must be expelled from the country (items adapted from the European Social Survey, 2014; α = 0.87).

Feelings of insecurity and the belief that women are more fragile than men formed the two predictors of support for expelling immigrants. First, two items measured participants’ feelings of insecurity. For personal insecurity, they had to indicate, on a scale from 1 = *totally safe* to 4 = *totally unsafe*, how they felt when walking alone in their neighborhood after dark (adapted from the European Social Survey, 2014). For collective insecurity, they indicated, on a scale from 1 = *not at all* to 5 = *a lot*, whether they feared an increase in violence and vandalism in their neighborhood (adapted from Sniderman et al., 2004). Because the correlation between these two items (*r* = 0.63, *p* < 0.001) can be qualified as large (Cohen, 1988), we computed a two-item score of feelings of insecurity (α = 0.76). Note that, because they had a different scale, we rescaled the two insecurity items to range from 0 to 10. Second, benevolent sexism (BS) was measured with 11 items (Glick and Fiske, 1996; French translation by Dardenne et al., 2006). Two of its subdimensions—PP (four items) and CGD scales (three items)—tap the belief that women are different from and more fragile than “strong” men, and thus need their protection. Preliminary exploratory factor analysis (EFA) revealed that these items loaded on the same factor, with the exception of one item (i.e., “in a disaster, women ought to be rescued before men”). For this reason we created a six-item score (PP–CGD: α = 0.81; see Glick and Fiske, 1996, for the items’ wording) that we differentiated from heterosexual intimacy (HI—four items loading on a second factor; α = 0.85). In addition, to single out the “strength vs. fragility” side of benevolent sexism from general sexist beliefs, we added as a control the score of hostile sexism (HS—all 11 items from Glick and Fiske, 1996; α = 0.94). For both benevolent and hostile sexism, response scales ranged from 1 = *totally disagree* to 7 = *totally agree*, with high scores indicating strong sexist beliefs.

### Results

Hierarchical regressions were used to test the outlined hypotheses. All continuous scores were centered at the grand mean. Main effects of the control and independent variables were entered in Model 1: experimental condition (1 = *Ivan condition*), gender (1 = *male*), age, education (1 = *high school diploma or higher*), having dual citizenship, feelings of insecurity, benevolent (PP–CGD and HI) and hostile sexist beliefs. The interactions between condition and BS: PP–CGD or feelings of insecurity were entered in Model 2. Then, we explored in further models whether there were differences between male and female participants, by entering the necessary two- and three-way interactions. Only significant three-way interactions were included in Model 3. Final models are presented in **Table 2**.

**Table 2:** Results of regression analyses, Experiment 1.

	Model 1			Model 2			Model 3		
	*b*	SE	β	*b*	SE	β	*b*	SE	β
Intercept	3.57	(0.23)		3.57	(0.23)		3.46	(0.23)	
Condition (C)	0.17	(0.18)	0.05	0.17	(0.18)	0.05	0.45^∗^	(0.23)	0.14
Male (M)	0.07	(0.20)	0.02	0.09	(0.19)	0.03	0.35	(0.27)	0.10
Age	-0.00	(0.01)	-0.01	-0.00	(0.01)	-0.00	-0.00	(0.01)	-0.01
Education	-1.03^∗∗∗^	(0.23)	-0.28	-1.00^∗∗∗^	(0.23)	-0.27	-1.03^∗∗∗^	(0.23)	-0.27
Dual citiz.	-0.13	(0.20)	-0.04	-0.18	(0.20)	-0.05	-0.18	(0.19)	-0.05
Insecurity (I)	0.23^∗∗∗^	(0.04)	0.34	0.29^∗∗∗^	(0.05)	0.43	0.30^∗∗∗^	(0.05)	0.45
BS: PP–CGD	0.06	(0.11)	0.04	-0.14	(0.14)	-0.09	-0.42^∗^	(0.17)	-0.28
BS: HI	0.21^∗∗^	(0.08)	0.18	0.22^∗∗^	(0.08)	0.18	0.19^∗^	(0.08)	0.16
HS	0.42^∗∗∗^	(0.10)	0.32	0.37^∗∗∗^	(0.10)	0.28	0.40^∗∗∗^	(0.10)	0.30
C × I				-0.08	(0.07)	-0.08	-0.10	(0.07)	-0.10
C × BS: PP–CGD				0.42^∗^	(0.18)	0.19	0.80^∗∗∗^	(0.23)	0.37
C × M							-0.59	(0.36)	-0.15
M × BS: PP–CGD							0.62^∗∗^	(0.23)	0.26
C × BS: PP–CGD × M							-0.81^∗^	(0.34)	-0.25
*R*^2^	62.0%			63.6%			66.5%		

*Unstandardized and standardized coefficients. ^∗^p < 0.05, ^∗∗^p < 0.10, ^∗∗∗^p < 0.001.*

As shown in Model 1, participants expressed a similar support for expelling immigrants after seeing the two political posters. Moreover, age, gender, and having an immigrant background did not have a significant impact. Consistent with previous research (e.g., Lancee and Sarrasin, 2015), those with a high school diploma or more expressed less support for expelling immigrants than those without. When it comes to the factors of interest here, feelings of insecurity were, as expected, positively related to an increased support for expelling immigrants. The belief that women are different and weaker than men (BS: PP–CGD) had no significant impact (note that hostile sexist beliefs and heterosexual intimacy were both related to a higher support). Then, the two interactions between the experimental condition and feelings of insecurity or benevolent sexist beliefs were entered in Model 2 (Δ*R*^2^, *p* = 0.056). Contrary to our expectations, the impact of feelings of insecurity was not moderated by the experimental condition (and this interaction was not further moderated by gender). In contrast, the impact of BS: PP–CGD was found to be different after exposure to the Ivan poster than to the Sheep poster.

**FIGURE 1:**
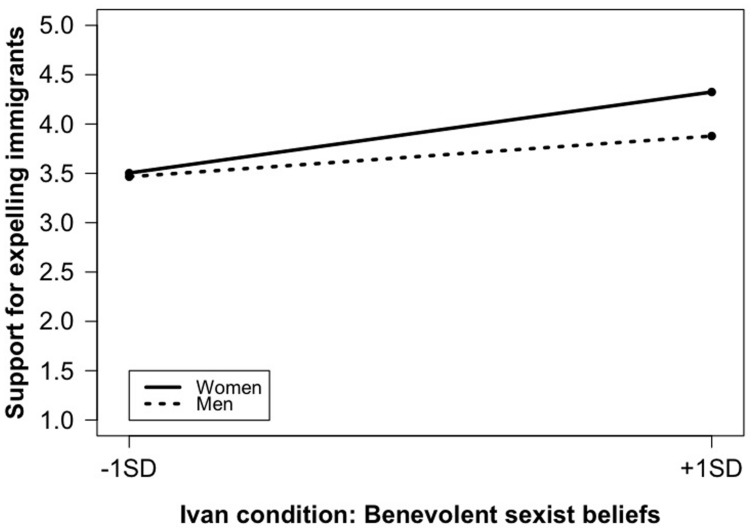
**Experiment 1: Benevolent sexism × participants’ gender on support for expelling immigrants; Ivan condition**.

**FIGURE 2:**
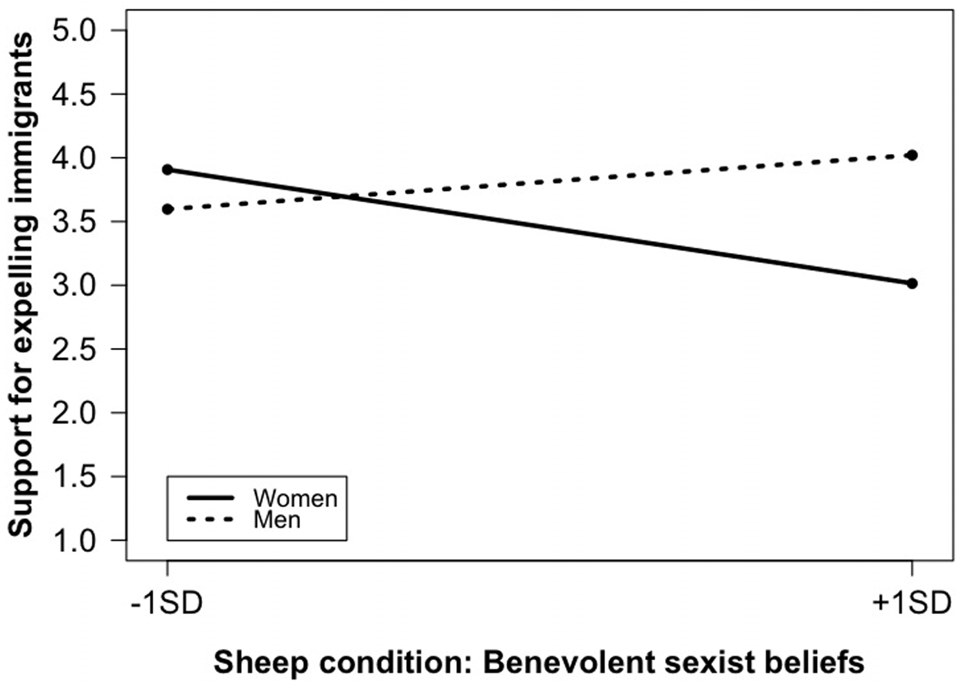
**Experiment 1: Benevolent sexism × participants’ gender on support for expelling immigrants; Sheep condition**.

We further verified whether participants’ gender qualified this interaction. To this purpose we entered in Model 3 the two necessary two-way interactions along with the three-way interaction (Δ*R*^2^, *p* = 0.02). The three-way interaction was found to yield a significant impact. Simple slopes estimation (Preacher et al., 2006) showed that benevolent sexist beliefs were related differently to support for expelling immigrants across conditions and genders. **Figure 1** reveals that, in the Ivan condition, among female participants, benevolent sexist beliefs were related to more support for expelling immigrants (*b* = 0.38, SE = 0.18, *p* = 0.03). A similar but non-significant trend was observed among male participants (*b* = 0.19, SE = 0.20, *p* = 0.33; note, however, that the difference between men and women scoring high in benevolent sexism was not significant: *b* = -0.44, SE = 0.38, *p* = 0.24). An opposite pattern was found in the Sheep condition (see **Figure 2**). Again, for male participants, the link was not significant (*b* = 0.20, SE = 0.19, *p* = 0.30). For female participants, however, the link was negative and significant (*b* = -0.42, SE = 0.17, *p* = 0.01). Point tests further revealed that there were no significant differences between men and women scoring low in benevolent sexism (-1 SD; *b* = -0.31, SE = 0.38, *p* = 0.42), whereas women scoring high in benevolent sexism expressed lower support for expelling immigrants than men with similar scores (+1 SD; *b* = 1.01, SE = 0.35, *p* = 0.005).

Finally, we ran models (omitted from Table 2) to verify whether the impact of education and heterosexual intimacy differed across the conditions. First, because students with higher educational attainment were found to be less sensitive to anti-immigrant posters (Matthes and Marquart, 2013; Schmuck and Matthes, 2014), we entered the interaction between education and the condition in an additional model. The impact of education was found not to vary across the two conditions (*b* = -0.01, SE = 0.44, *p* = 0.97). Second, we examined whether, as assumed, the impact of the third component of benevolent sexism—i.e., heterosexual intimacy—was not activated by sexual threat cues. To this end, we added to the models presented in **Table 2** the following interactions: HI × condition, HI × gender, and HI × condition × gender. Results showed that the impact of HI did not differ across the two conditions (*b* = -0.00, SE = 0.15, *p* = 0.98; Model 2), and that this interaction was not further moderated by gender (*b* = 0.03, SE = 0.31, *p* = 0.93; Model 3). Importantly, the three-way interaction between condition, gender, and BS: PP–CGD remained significant when adding these controls.

### Discussion

Experiment 1 compared reactions when participant were faced with two anti-immigrant political posters that had been used during the same Swiss political campaign. These posters depicted immigrants as a general and non-specific threat or as a sexual threat, respectively. As expected, feelings of insecurity were related to a greater support for expelling criminal immigrants. However, contrary to our expectations, their impact did not vary across conditions or genders. The belief that women are different from and more fragile than men was related to more negative attitudes toward immigrants in the sexual violence cues conditions, but, contrary to our hypothesis, only among female participants. As outlined below, a second experiment was designed to shed further light on these findings.

The impact of feelings of insecurity may not have differed across the two conditions because both contained a threatening representation of immigrants, that is, in the rapist poster condition, as a danger to women, and in the sheep poster condition, as deviants needing to be expelled from the country. Moreover, it may be that the impact of physical violence cues is greater when immigrants are presented as physically—and not only sexually—violent and dangerous. For these two reasons, Experiment 2 contains, in addition to a rapist condition, a control condition with no poster and posters picturing male immigrants as a danger to the local population (i.e., as is the case with violent criminals or, to some degree, also with drug dealers, often associated with insecurity in certain neighborhoods or parts of town). Note also that we could not treat feelings of personal and collective insecurity separately, which may explain why they did not have a stronger impact in women (we expected this to be the case only for feelings of personal insecurity). This empirical closeness may have been due to both measures containing a reference to insecurities at the neighborhood level (i.e., walking alone in the neighborhood, and fear of vandalism and violence in the neighborhood). For this reason, we use more distinct measures in Experiment 2. First, to assess feelings of personal insecurity, we use an item tapping the fear of unknown men, immigrant or not. Adjusting our initial hypothesis, we expect that in particular women who fear unknown men will react with anti-immigrant attitudes when exposed to the rapist poster, compared to the three other conditions. Second, to measure feelings of collective insecurity, we consider fear of violence and vandalism at two different societal levels, that is the neighborhood and the nation. Because these fears relate to societal stability, perceived as being endangered by criminal immigrants, we expect that both men and women with such feelings will react with negative attitudes when exposed to a poster associating immigrants to crimes (rape, violence, or drug trafficking), compared to those with similar feelings who encounter no poster.

For male participants the belief that women are more fragile than men was unrelated to support for expelling criminal immigrants in both of the two conditions. In the sexual threat cues condition, benevolent sexism (i.e., the PP and CGD subdimensions) was significantly related to anti-immigrant stances only among the female participants. However, with the data at hand, it is not possible to determine whether this was provoked by the sexual component of the threatening message, or by its representation of a man as physically dangerous. Comparing the impact of posters picturing male immigrants associated with different types of crimes will help tackle this question. On the one hand, women who believe in the protective role of men may react with harsher anti-immigrant attitudes when presented with a dangerous male immigrant, he being either sexually or generally violent. If so, women with benevolent sexist beliefs should express heightened anti-immigrant stances when exposed to the rapist or violent criminal depictions, in comparison to the drug dealer or no-poster conditions. On the other hand, it may be that female participants reacted more strongly to the Ivan poster because women are more often victims of sexual aggression. In line with this argument, fear of being raped was found to underlie negative attitudes toward out-group men more strongly than attitudes toward in-group men (Navarrete et al., 2010). Another related interpretation may be that benevolent sexism has a different meaning for men and women. Sibley and Perry (2010) suggested that the original definition of benevolent sexism—paternalistic views toward women—holds for men only. In the case of women, benevolent sexism in fact reflects a positive view of the in-group (i.e., women are better than men), especially when hostile sexism (that is, negative and harsh attitudes) is controlled for. Indeed, these authors found that among men, benevolent sexism was related to negative attitudes toward gender equality (as is generally expected), while among women it predicted positive attitudes. Following this rationale, women scoring high in benevolent sexism may react to the Ivan poster because they feel more connected to women in general, the alleged victims of (the fictitious) Ivan. Based on this reasoning, women with strong benevolent sexist beliefs should express heightened anti-immigrant stances only when exposed to the rapist depiction, but not in the violent criminal, drug dealer, or no-poster conditions.

The reversed effect found in the Sheep-poster, that is, women showing a decreasing tendency toward the expulsion of immigrants, also requires attention. The sheep-poster may indeed have conveyed multiple messages. While a black sheep certainly refers to the outsider-status of immigrants, the fact that it was violently kicked out by a white sheep may have triggered empathy and concern among women high in benevolent sexism. Indeed, while women holding benevolent sexist beliefs may be more sensitive to cues of sexual violence targeting the safety of their own in-group, when confronted with aspects of immigrants they can relate to, such as their need for protection, solidarity may be triggered, leading to more inclusive attitudes.

Finally, while Experiment 1 revealed an impact of the posters used, we cannot ascertain that this was due to the mere exposure to their content. Indeed, these posters were widely known in Switzerland, as reflected in almost all participants being familiar with them. Thus, the findings may have been provoked by a reminder of the heated debates that followed their publication rather than to their content itself. To exclude this, Experiment 2 was conducted in a comparable national context but in which posters of this kind had not been used. Germany was selected for its similarity to Switzerland with respect to its immigration history (through the massive arrival during the 20th century of *Gastarbeiter*, i.e., migrant workers), its citizenship regime (based on an ethnic conception of the nation), and the salience of heated debates on immigration and cultural diversity.

## Experiment 2

### Method

#### Participants

Data were collected through a large social networking service, in which a randomly placed advertisement invited German residents to take part in an online survey on “German society.” Eight hundred and forty-two people clicked on the link, and 218 completed the questionnaire. We selected data of German citizens (*n* = 181; 97 women and 84 men). Participants ranged in age from 18 to 75 (*M* = 34.18, SD = 12.20; note that two missing data were replaced with the mean). Twenty-three participants reported having another national citizenship. Finally, around two thirds of the sample (*n* = 123) declared having started or completed higher education (note that the response options of the educational attainment scale were adapted to the German context and thus slightly differed from those used in Experiment 1).

#### Procedure

The overall procedure was similar to Experiment 1. Participants first completed a series of scales on different societal topics. Next, they read that immigration—the topic of the second part of the question—is a much-debated issue in many European countries, and that political parties often rely on posters to express their opinions on this matter. Participants were then randomly assigned by Psychsurveys to one of four conditions. In the *No poster* condition (*n* = 45) no further information was added. In the three other conditions, two extra sentences described a type of poster they could potentially see in Germany. To our knowledge, so far no poster associating, akin to the Ivan poster, male immigrants and sexual or physical violence, has been used in Germany. In all cases the poster—which we created ourselves—showed a frontal picture of a bearded white man in his 20 s, with “Igor B.” written in a black bar hiding his eyes. A black square, in front of his torso, contained the description of a criminal followed by “soon naturalized?” The different types of criminal, constituting the only difference across the three last conditions, were: *Rapist* (*n* = 44), *Violent criminal* (VC, *n* = 52), and *Drug dealer* (DD, *n* = 40). Then, as in Experiment 1, participants responded to various measures related to immigration.

Experiment 2 followed the recommendations of the American Psychological Association. The introduction to the questionnaire was the same as in Study 1. After completing the questionnaire, participants were debriefed that the young man presented in the poster was not an actual criminal.

#### Measures

The whole questionnaire was in German. Means, SD, and intercorrelations are displayed in **Table 1**. As in Experiment 1, the dependent variable was measured by the same three items evaluating participants’ support for expelling law- and norm-breaking immigrants (α = 0.88).

As in Experiment 1, feelings of insecurity and benevolent sexist beliefs formed our independent variables of interest. First, we asked participants to what extent (from 1 to 4) they felt safe when encountering a group of unknown men at night. Then, two items measured participants’ feelings of collective insecurity: they stated to what extent (from 1 to 7) they feared an increase in violence and vandalism in their neighborhood or in Germany (adapted from Sniderman et al., 2004). While the two collective insecurity items were strongly correlated (*r* = 0.66, α = 0.79), they were only moderately related to fear of unknown men (neighborhood: *r* = 0.43; country: *r* = 0.31). For this reason we considered them as two different predictors. Finally, the belief that women are different from and more fragile than men was tapped with the same six items as in Experiment 1 (PP and CGD; α = 0.82), which were shown by EFA to load on the same dimension (loadings > 0.30). As in Experiment 1, heterosexual intimacy (α = 0.88) and hostile sexist beliefs (α = 0.93) were used as control variables (for both benevolent and HS, German translation by Eckes and Six-Materna, 1999).

### Results

As in Experiment 1, hierarchical regressions were used to test the outlined hypotheses. All continuous scores were centered at the grand mean. Dummy variables tapping the experimental conditions (with the no-poster condition as reference category) were entered in Model 1, along with gender (1 = *male*), age, education (1 = *having started or completed higher education*), dual citizenship, fear of unknown men, feelings of collective insecurity, benevolent (PP–CGD and HI) and hostile sexist beliefs. The interactions between the conditions and PP–CGD or feelings of collective insecurity were entered in Model 2 (preliminary analyses revealed that the impact of fear of unknown men did not vary significantly across the conditions or across genders; these interactions were thus not entered in Model 2). Finally, we verified whether the significant two-way interactions were further moderated by participants’ gender. As no significant three-way interactions were revealed, they were excluded from the final model. Final models are presented in **Table 3**.

**Table 3:** Results of regression analyses, Experiment 2.

	Model 1			Model 2		
	*b*	SE	β	*b*	SE	β
Intercept	3.56	(0.31)		3.11	(0.30)	
*Ref: control*				
Rapist (R)	-0.09	(0.31)	-0.02	-0.06	(0.30)	-0.01
Violent criminal (VC)	-0.27	(0.30)	-0.07	-0.23	(0.30)	-0.06
Drug dealer (DD)	-0.40	(0.34)	-0.08	-0.42	(0.33)	-0.09
Male	0.60^∗^	(0.24)	0.16	0.50^∗^	(0.24)	0.13
Age	0.01	(0.01)	0.08	0.02	(0.01)	0.09
Education	-0.14	(0.25)	-0.04	-0.30	(0.25)	-0.07
Dual citizenship	0.14	(0.34)	0.03	0.12	(0.33)	0.02
Insecurity (I-P)	0.06	(0.17)	0.02	0.06	(0.17)	0.03
Insecurity (I-C)	0.47^∗∗∗^	(0.09)	0.41	0.22	(0.15)	0.19
BS: PP–CGD	0.15	(0.13)	0.10	-0.00	(0.18)	-0.00
BS: HI	0.07	(0.09)	0.07	0.07	(0.08)	0.06
HS	0.36^∗∗^	(0.13)	0.22	0.33^∗∗^	(0.13)	0.20
R × I-C				0.06	(0.21)	0.02
VC × I-C				0.51^∗∗^	(0.19)	0.23
DD × I-C				0.35†	(0.20)	0.16
R × BS: PP–CGD				0.58^∗^	(0.26)	0.18
VC × BS: PP–CGD				0.11	(0.25)	0.03
DD × BS: PP–CGD				0.00	(0.26)	0.00
*R*^2^	48.3%			52.9%		

*Unstandardized and standardized coefficients. †p < 0.10, ^∗^p < 0.05, ^∗∗^p < 0.10, ^∗∗∗^p < 0.001.*

Model 1 revealed that there was no significant main effect of the experimental condition: participants in the Rapist, VC, and DD conditions did not express a higher support for expelling immigrants compared to the No-poster condition. Men were keener to expel immigrants, whereas age, education, and dual citizenship had no significant effect. While feelings of personal insecurity (i.e., fear of unknown men) yielded no significant impact, feelings of collective insecurity significantly predicted a stronger support for expelling immigrants. In addition, benevolent sexist beliefs (both PP–CGD and HI) were not related to support for expelling immigrants (note that, in contrast, HS had a significant impact).

**FIGURE 3:**
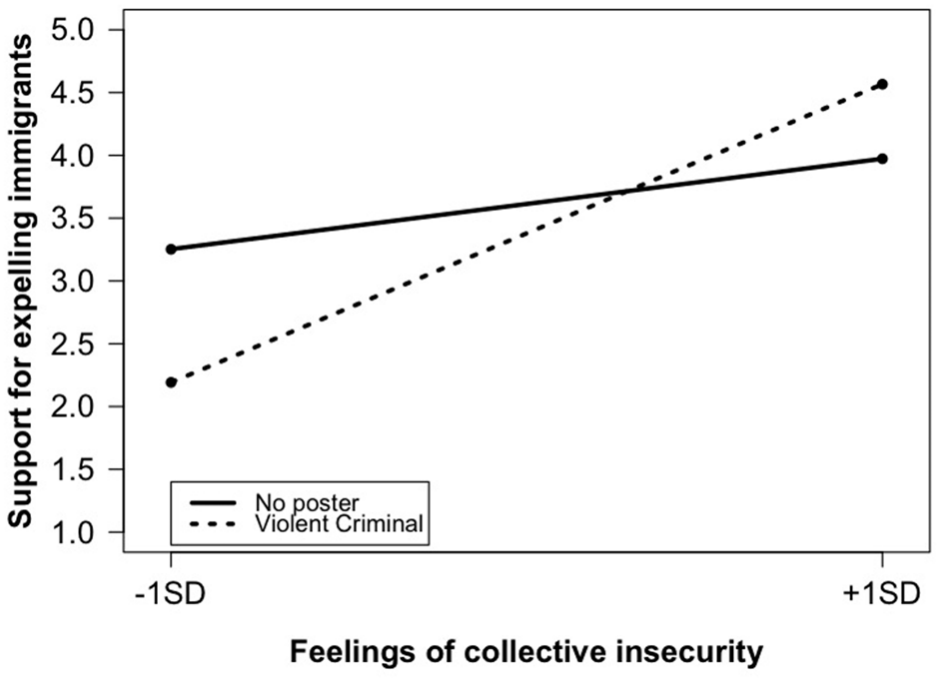
**Experiment 2: Feelings of collective insecurity on support for expelling immigrants; No poster vs. Violent criminal (VC) conditions**.

Entering the six interaction terms in Model 2 significantly increased the explained variance (Δ*R*^2^, *p* = 0.03). First, the impact of feelings of collective insecurity was found to be different in the VC and DD (*p* = 0.08) conditions than in the control condition. In contrast, the interaction term involving the Rapist condition yielded no significant effect. As shown in **Figure 3**, feelings of collective insecurity were significantly related to an increased support for expelling immigrants in the VC condition (*b* = 0.73, SE = 0.14, *p* < 0.001), while this was not the case in the No-poster condition (*b* = 0.22, SE = 0.15, *p* = 0.13). Point test further showed that the effect was mostly due to individuals with low feelings of insecurity (-1 SD) expressing a lower support for expelling immigrants in the VC condition (*b* = -1.06, SE = 0.41, *p* = 0.01), compared to those with similar feelings in the No-poster condition. The difference between the two conditions for participants who reported high feelings of insecurity (+1 SD) was not significant (*b* = 0.59, SE = 0.45, *p* = 0.19). Note, however, that if we consider participants with very strong feelings of insecurity (+2 SD), those in the VC condition reported a stronger support for expelling immigrants than in the control condition (*b* = 1.44, SE = 0.72, *p* = 0.05). A similar—but less strong—pattern was revealed for the DD vs. No-poster conditions. Feelings of collective insecurity were positively related to support for expelling immigrants in the DD condition (*b* = 0.78, SE = 0.25, *p* = 0.002). However, point tests revealed that participants reporting low (-1 SD; *b* = -0.42, SE = 0.33, *p* = 0.20) or strong (+1 SD; *b* = -0.08, SE = 0.37, *p* = 0.83) feelings of collective insecurity did not differ in their support of expelling immigrants across the two conditions.

**FIGURE 4:**
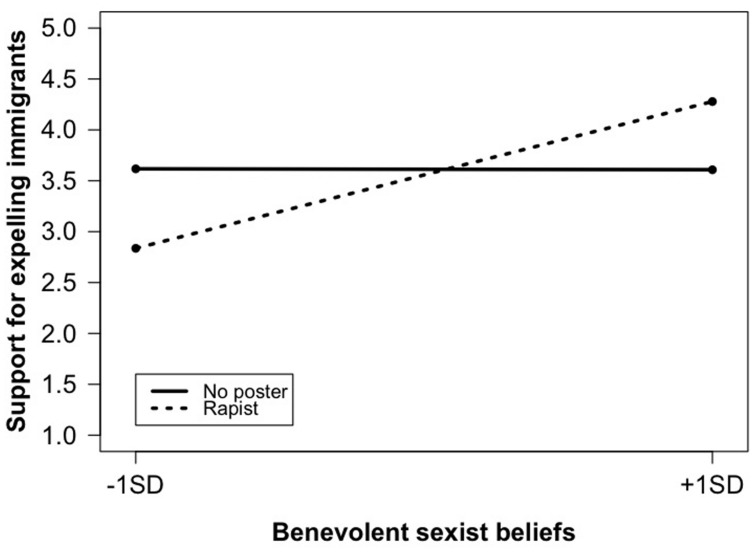
**Experiment 2: Benevolent sexist beliefs on support for expelling immigrants; No poster vs. Rapist conditions**.

The impact of BS: PP–CGD did not vary across the VC, the DD, and the No-poster condition. In contrast and in line with the expectation that benevolent sexism drives immigration attitudes only when sexual cues are present, the interaction involving the Rapist condition reached significance. As in the case of feelings of insecurity and the VC representation, the Rapist representation appeared to polarize attitudes among those scoring low vs. high in BS: PP–CGD. Simple slopes estimation (see **Figure 4**) indeed revealed that benevolent sexist beliefs were positively related to an increased support for expelling immigrants in the Rapist condition (*b* = 0.58, SE = 0.21, *p* = 0.007), while there was no significant relationship between these two variables in the No-poster condition (*b* = -0.00, SE = 0.18, *p* = 0.98). Point tests further showed that individuals with high benevolent sexist beliefs (+1 SD) did not differ in their support for expelling immigrants across the two conditions (*b* = 0.67, SE = 0.45, *p* = 0.13), while individuals with low benevolent sexist beliefs (-1 SD) confronted with the Rapist poster expressed a marginally significantly lower support than those with similar beliefs in the No-poster condition (*b* = -0.78, SE = 0.44, *p* = 0.08). When considering participants with more extreme scores (±2 SD), those scoring low in BS: PP–CGD expressed less anti-immigrant stances in the Rapist than in the No-poster condition (*b* = -1.51, SE = 0.70, *p* = 0.03), while those scoring highly expressed more negative attitudes (*b* = 1.40, SE = 0.71, *p* = 0.05).

Finally, as in Experiment 1, additional models were conducted to examine whether the impact of education and heterosexual intimacy varied across the four experimental conditions. First, the impact of education could be considered as identical across the conditions (*p* > 0.45). Another set of additional models further showed that also the impact of heterosexual intimacy did not vary across the four experimental conditions (*p* > 0.40).

### Discussion

Experiment 2 allowed both testing the impact of more fine-grained measures of feelings of security and comparing different threatening representations of a male immigrant. Moreover, by conducting an experiment in a national setting with no well-known posters containing sexual threat cues, we tested the impact of a mere exposure to these posters. Extending the results obtained with Experiment 1, Experiment 2 showed that depictions of male immigrants as violent but not sexually threatening resonated with different antecedents of anti-immigrant attitudes than those of male immigrants as a rapist. Moreover, rather than only activating negative attitudes among those predisposed to be sensitive to such cues, depictions of male immigrants as having committed diverse crimes appeared to polarize attitudes among German citizens. These polarized reactions (i.e., more positive or more negative attitudes toward immigrants) explain why support for expelling immigrants, on average, was not stronger in the conditions including a poster than in the No-poster condition in Experiment 2.

First, a depiction of a male immigrant as a violent criminal (and to a lesser extent, as a drug dealer) moderated the impact of feelings of collective insecurity, while the rapist poster did not do so. Thus, particularly depictions of male immigrants as a danger to the whole national society—by being extremely violent or by distributing illegal drugs—resonate with the extent to which citizens fear for the security in their neighborhood and country. This may explain why no interaction between the feelings of insecurity and the rapist condition was found in Experiment 1. It should be noted, however, that in Experiment 2 the moderating impact of physical violence cues was mostly due to those who do not feel insecure expressing an adverse or reactance reaction when presented with the violent criminal poster: they supported less the expelling of immigrants than those with similar feelings of (in)security in the No-poster condition. This is similar to the effect found by Matthes and Marquart (2013): when presented with a (too) strong and obvious threat message, those not predisposed to reject immigration (i.e., with high educational attainment or low feelings of collective insecurity) may express a marked disagreement with the message’s content. Finally, in line with our expectation, those who reported feeling extremely worried reacted with harsher attitudes.

Second, and contrary to previous research (Chandler and Tsai, 2001; Rustenbach, 2010), Experiment 2 revealed no impact of personal feelings of insecurity on attitudes toward immigrants. This result is, however, in line with those of Vecchione et al. (2012), who showed that when feelings of both personal and collective insecurity are used simultaneously to predict attitudes toward immigrants, the former make no unique contribution to explaining attitudes. Thus, those who fear for their personal security may be more negative toward immigrants because they are also worried about security at a more collective level, but the reverse may not be true: some may feel worried about society’s stability and harmony in general without feeling to be personally at risk. This may be the case of individuals living in rural areas, where the crime rate is generally lower than in large cities but where fears of immigrants and violence tend to be common.

Finally, the analyses confirmed that, in particular, the gendered dimension of presenting a male immigrant as a rapist resonates with the belief that women are different and more fragile than men. However, in contrast to Experiment 1, Experiment 2 revealed no moderation of this effect by participants’ gender. This may have been because posters used in political campaigns (as those used in Experiment 1) are more strongly associated with the environment in which they were depicted (that is, streets in Switzerland). These associations with one’s personal environment (i.e., neighborhood, town) could potentially lead to an increased realistic fear in women of being personally confronted with a violent immigrant, or, to identifying with other women from the community who could potentially become a victim of a violent immigrant. Moreover, because female participants in Experiment 1 were familiar with the Ivan poster (to which they were regularly exposed during the Swiss political campaign on the expulsion of immigrants), they (or at least some of them) may have had time to build an association between male immigrants and sexual danger. This may indicate that repeated exposure to threatening material has a stronger impact on individuals’ attitudes than a one-off exposure to it.

## General Discussion

Building on previous research on sensitivity to threat cues, the findings of the two studies conducted in different national settings showed that individuals react quite differently to threatening depictions of immigrants. First, as expected, attitudes toward immigrants were more strongly predicted by benevolent sexist beliefs when individuals (women in Experiment 1) were exposed to sexual threat cues than when they were not. Then, Experiment 2 highlighted similar mechanisms in the case of feelings of insecurity and depictions of male immigrants as violent criminals. Importantly, the revealed moderation effects appeared to be due both to reactance and activation mechanisms, which explain the lack of main effects of the experimental conditions. On the one hand, individuals who were quite unlikely to “buy into” the messages conveyed by the posters expressed more positive attitudes. This is akin to the backfire or contrast effects found in political campaigning: Individuals tend to shift away from the message they are presented, for instance when the source of the message is despised (Aaroe, 2012; Bechtel et al., 2015). On the other hand, in line with social psychological research on the activating power of threat cues (Thomsen et al., 2008; Duckitt and Sibley, 2010), those expressing security concerns and those who believed in the protective role of men were slightly more willing to expel immigrants after exposure to political posters containing threat cues.

Beyond the impact of threatening cues, the present experiments shed light on the complex nature of benevolent sexism and its relationship with more blatant forms of prejudice. First, our results suggest that, instead of using the benevolent sexism scale as a whole, it is important to examine the impact of its subdimensions. We indeed found, as hypothesized, that the subdimensions representing women as fragile and men as strong protectors resonated with sexual threat cues, while the impact of heterosexual intimacy did not differ across the conditions. Second, as expected, in the two experiments benevolent sexism correlated moderately with anti-immigrant stances. However, when hostile sexism and the other independent variables were considered, except for one case their impact became non-significant. Confirming previous research, this result indicates that the protective side of benevolent sexism as such may not be related to blatant and open forms of prejudices. Yet, there are other ways by which exposure to benevolent sexism can have negative consequences for gender equality and women’s well-being (e.g., women’s lower willingness to take part in collective action, Becker and Wright, 2011; lower condom use, Fitz and Zucker, 2015). In line with this research, our findings confirm that benevolent sexism operates through pathways other than blatant discrimination to contribute to the maintenance of inequalities between social groups.

Finally, while the findings of the present research undoubtedly contribute to understanding the impact of threat cues, they certainly have limitations that need to be addressed in future research. First, we measured only very short-term reactions, and have no means of knowing whether individuals’ willingness to expel immigrants was affected in the long term. There is, however, indication that this would be the case: longitudinal analyses have shown that being confronted with anti-immigrant political advertising generates negative affects, which, in turn, increase attention to political messages (Schemer, 2012). Thus, the initial negative reaction provoked by an anti-immigrant poster among individuals not predisposed to prejudice may have consequences on a longer term, by drawing their attention to subsequent anti-immigrant advertising. Second, it is known that contextual circumstances perceived as threatening (e.g., strong presence of immigrant minorities) also shape the impact of antecedents of anti-immigrant stances (e.g., feelings of insecurity). For this reason, future research on sensitivity to anti-immigrant posters should also take into account where respondents live, and what they experience in their everyday lives. It may be that those who live in neighborhoods with a strong presence of immigrants but who feel safe are “immune” to the messages contained in anti-immigrant posters, because the representations conveyed do not match their everyday experience. In contrast, the anti-immigrant attitudes of those living in the same neighborhoods but who feel unsafe may be strongly activated by depictions associating immigrants to violence.

## Conflict of Interest Statement

The authors declare that the research was conducted in the absence of any commercial or financial relationships that could be construed as a potential conflict of interest.
